# Supplementary material for: Synthesis and Characterisation of Poly(3-hydroxybutyrate-co-3-hydroxyvalerate)-b-poly(3-hydroxybutyrate-co-3-hydroxyvalerate) Multi-Block Copolymers Produced Using Diisocyanate Chemistry
Source: Polymers (Basel). 2023 Jul 31;15(15):3257. doi: 10.3390/polym15153257 (PMC10422281; doi:10.3390/polym15153257)
Supplement: Supplementary file 1 [file polymers-15-03257-s001.zip › polymers-2505013_Supplementary material_final version.pdf]

Supporting Information

Synthesis and Characterisation of Poly(3-hydroxybutyrate-*co*-3-hydroxyvalerate)-*b*-poly(3-hydroxybutyrate-*co*-3-hydroxyvalerate) Multi-block Copolymers Produced Using Diisocyanate Chemistry

Jingjing Mai, Steven Pratt, Bronwyn Laycock*, and Clement Matthew Chan*

School of Chemical Engineering, The University of Queensland, Brisbane, QLD 4072, Australia

jingjing.mai@uq.edu.au (J.M.); s.pratt@uq.edu.au (S.P.)

* E-mail address: b.laycock@uq.edu.au (B.L.); c.chan@uq.edu.au (C.M.C.)

Table of Contents

SI.1. Details of hydroxy-functionalised PHBV random copolymers used 3
SI.2. Chemical shifts of ^{1}H-NMR spectra of reaction products of HDI with hydroxy-functionalised PHBV 4
SI.3. Chemical shifts of solid-state ^{13}C-NMR spectra of reaction products of HDI with hydroxy-functionalised PHBV 5
SI.4. DSC thermogram 6

SI.1. Details of hydroxy-functionalised PHBV random copolymers used

Table S1 Details of hydroxy-functionalised PHBV random copolymers used

	Material	3HV (mol%)	M_n (kDa)	M_w (kDa)	$Đ$	Concentration of end groups (mmoles/g PHA)				Thermal properties							
						[1°OH_{total}] *^{31}P-NMR | ^{1}H-NMR*	[2°OH_{total}] *^{31}P-NMR | ^{1}H-NMR*	[COOH] *^{31}P-NMR | ^{1}H-NMR*	[Crotonic] *^{31}P-NMR | ^{1}H-NMR*	Under 110 °C T_m (°C)	Under 110 °C ΔH_m (J/g)	Over 110 °C T_m (°C)	Over 110 °C ΔH_m (J/g)	Total ΔH_m (J/g)	T_c (°C)	ΔH_c (J/g)	T_g (°C)
Used to synthesize block copolymers	Random _1HV#4	1	28	51	1.8	0.032 | 0.027	0.052 | 0.031	0.021 | 0.014^	0.003* | 0.002	n.d.	n.d.	149.1/ 168.7	149.8	105.6	73.6	87.1	2.5
	Random _1HV#5	1	25	44	1.7	0.048 | 0.041	0.055 | 0.041	0.024 | 0.018^	0.004* | 0.003	n.d.	n.d.	124.2/ 169.5	118.6	118.6	83.6	43.7	1.8
Used to compare material properties with synthesized block copolymers	Random _1HV#1	1	86	186	2.2					n.d.	n.d.	157.6/ 174.0	102.3	102.3	98.3	87.1	2.6
	Random _1HV#2	1	117	273	2.3					n.d.	n.d.	160.0/ 175.0	102.5	102.5	103.4	89.2	2.6
	Random _1HV#3	1	194	455	2.3					n.d.	n.d.	159.5/ 170.0	106.0	106.0	104.7	87.3	4.0

SI.2. Chemical shifts of ^{1}H-NMR spectra of reaction products of HDI with hydroxy-functionalised PHBV

Table S2 Chemical shifts of ^{1}H-NMR spectra of reaction products of HDI with hydroxy-functionalised PHBV

Material	Proton label	Functional groups	Chemical shifts (ppm)
polymer end group containing secondary OH	$2°OH_{HB}$	1H, **H**O-CH(CH_3)-CH_2-CO-	3.00 -3.15
	$2°OH_{HV}$	1H, **H**O-CH(CH_2-CH_3)-CH_2-CO-	
	$H1_{HB}$	1H, HO-C**H**(CH_3)-CH_2-CO-	4.17
	$H1_{HV}$	1H, HO-C**H**(CH_2-CH_3)-CH_2-CO-	3.90
polymer end group containing primary OH	Et_1	2H, -O-C**H**$_2$-CH_2-OH	4.21
	Et_2	2H, -O-CH_2-C**H**$_2$-OH	3.78
	1°OH	2H, -O-CH_2-CH_2-O**H**	-
Protons of urethane containing end groups formed from reaction of hydroxyl groups with hexyl isocyanate	Ur_1	1H, -O-CH_2-CH_2-O-CO-N**H**-CH_2-CH_2-CH_2-CH_2-CH_2-CH_2-	4.92
	Ur_2	1H, -CO-CH_2-CH(CH_3)-O-CO-N**H**-CH_2-CH_2-CH_2-CH_2-CH_2-CH_2-	4.89
	Ur_3	2H, -O-CO-NH-C**H**$_2$-CH_2-CH_2-CH_2-CH_2-CH_2-	3.16
	Ur_4	2H, -O-CO-NH-CH_2-C**H**$_2$-CH_2-CH_2-CH_2-CH_2-	1.48
	Al_1	2H, -O-CO-(R)N-CO-NH-C**H**$_2$-CH_2-CH_2-CH_2-CH_2-CH_2-	3.22

SI.3. Chemical shifts of solid-state ^{13}C-NMR spectra of reaction products of HDI with hydroxy-functionalised PHBV

Table S3 Chemical shifts of PHBV materials in solid-state ^{13}C-NMR spectra

#	Functional groups	Chemical shifts (ppm)
HB_1	-O-CH(CH$_3$)-CH$_2$-**C**O-	160-180
HV_1	-O-CH(CH$_2$-CH$_3$)-CH$_2$-**C**O-	
HV_3	-O-**C**H(CH$_2$-CH$_3$)-CH$_2$-CO-	69
HB_3	-O-**C**H(CH$_3$)-CH$_2$-CO-	66
HB_2	-O-CH(CH$_3$)-**C**H$_2$-CO-	40
HV_2	-O-CH(CH$_2$-CH$_3$)-**C**H$_2$-CO-	38
HV_4	-O-CH(**C**H$_2$-CH$_3$)-CH$_2$-CO-	26
HB_4	-O-CH(**C**H$_3$)-CH$_2$-CO-	19
HV_5	-O-CH(CH$_2$-**C**H$_3$)-CH$_2$-CO-	8
Al_2	-O-CO-(R)N-CO-NH-CH$_2$-CH$_2$-**C**H$_2$-**C**H$_2$-**C**H$_2$-**C**H$_2$-	28
C_1	-O-CO-(R)N-**C**O-NH-CH$_2$-	142-160

SI.4. DSC thermogram

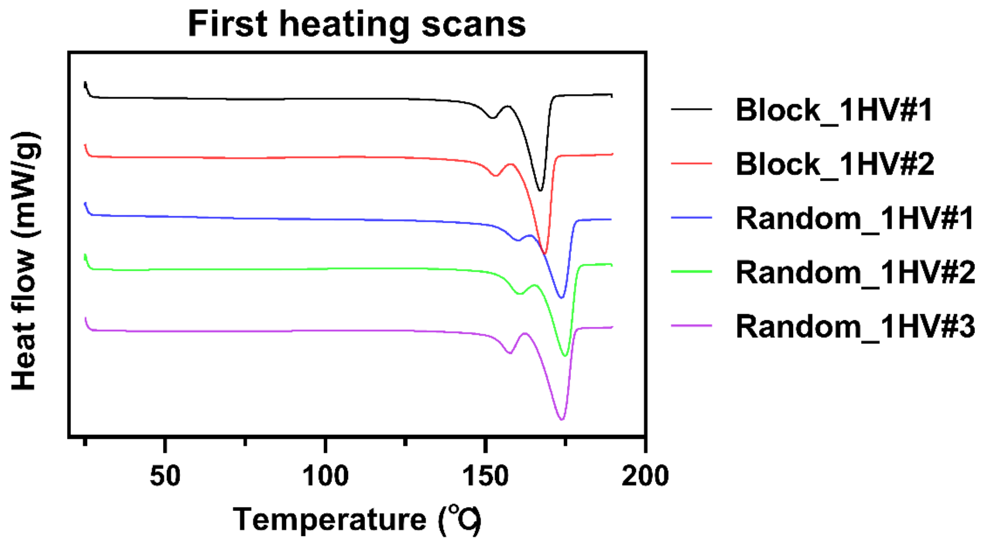

Fig. S1 First heating scans of 1 mol% 3HV block and random copolymers and high 3HV block, blend and random copolymers.

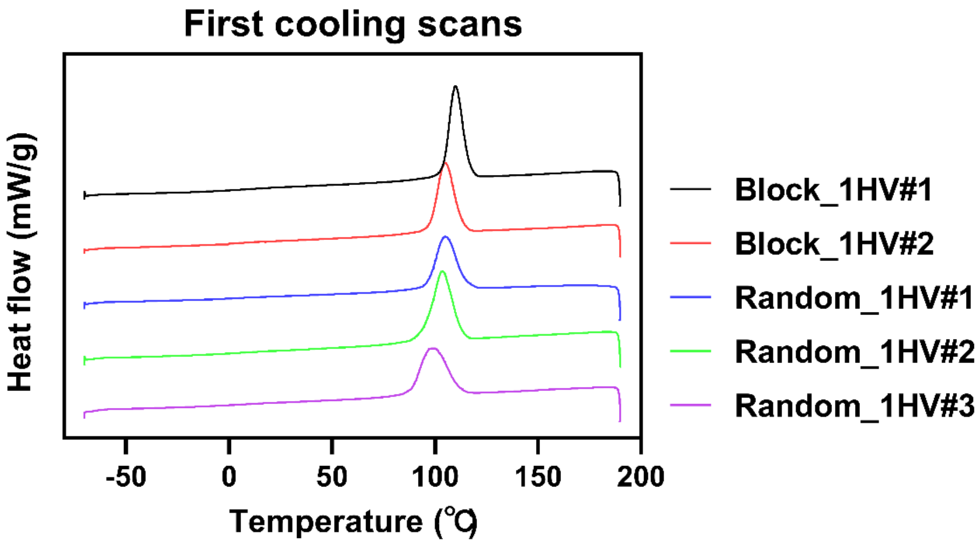

Fig. S2 First cooling scans of 1 mol% 3HV block and random copolymers and high 3HV block, blend and random copolymers.

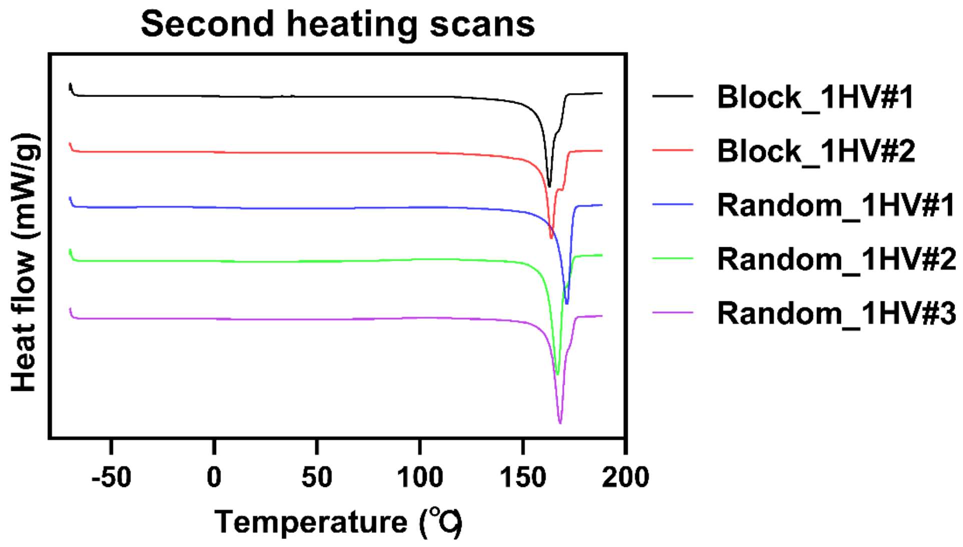

Fig. S3 Second heating scans of 1 mol% 3HV block and random copolymers and high 3HV block, blend and random copolymers.

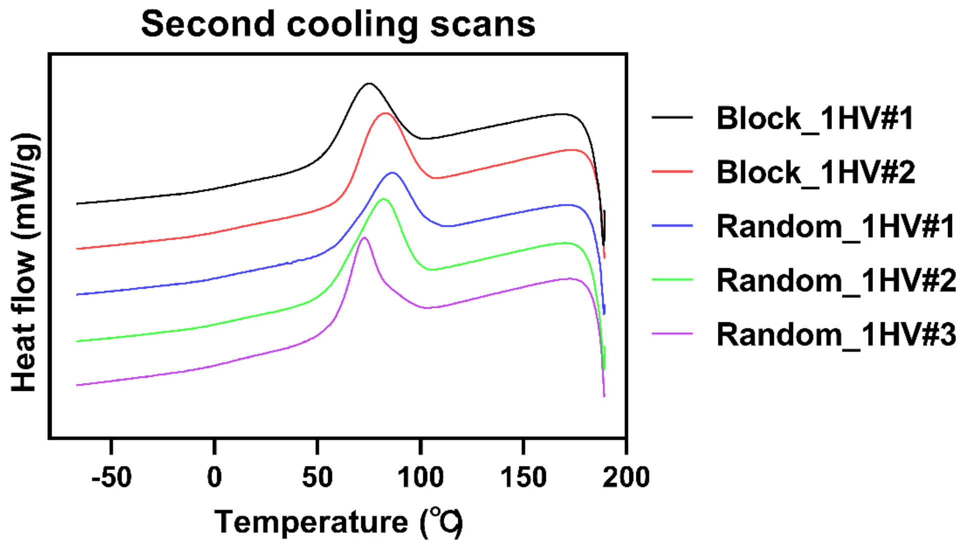

Fig. S4 Second cooling scans of 1 mol% 3HV block and random copolymers and high 3HV block, blend and random copolymers.

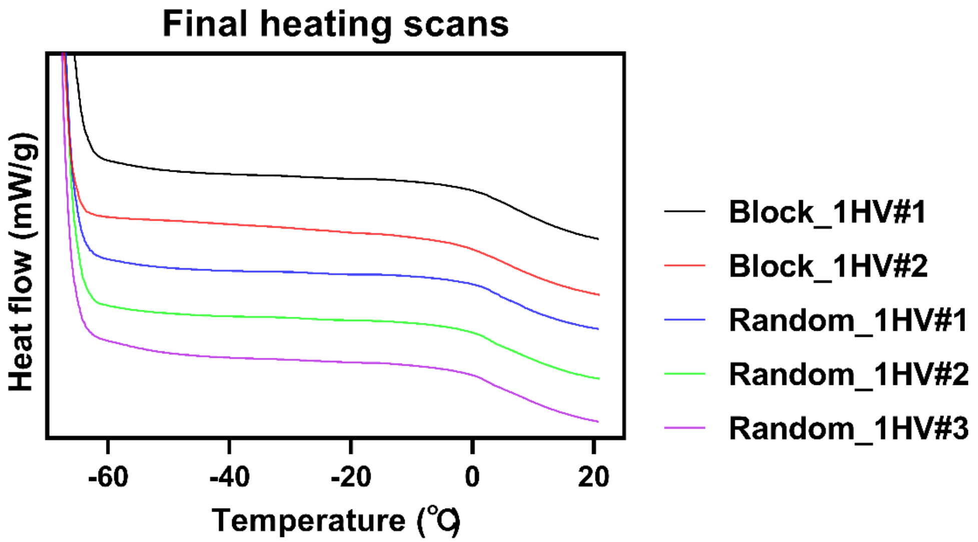

Fig. S5 Last heating scans of 1 mol% 3HV block and random copolymers and high 3HV block, blend and random copolymers.
